# Supplementary figures and images for: het-B allorecognition in Podospora anserina is determined by pseudo-allelic interaction of genes encoding a HET and lectin fold domain protein and a PII-like protein
Source: PLoS Genet. 2024 Feb 12;20(2):e1011114. doi: 10.1371/journal.pgen.1011114 (PMC10890737; doi:10.1371/journal.pgen.1011114)

## Slide 1
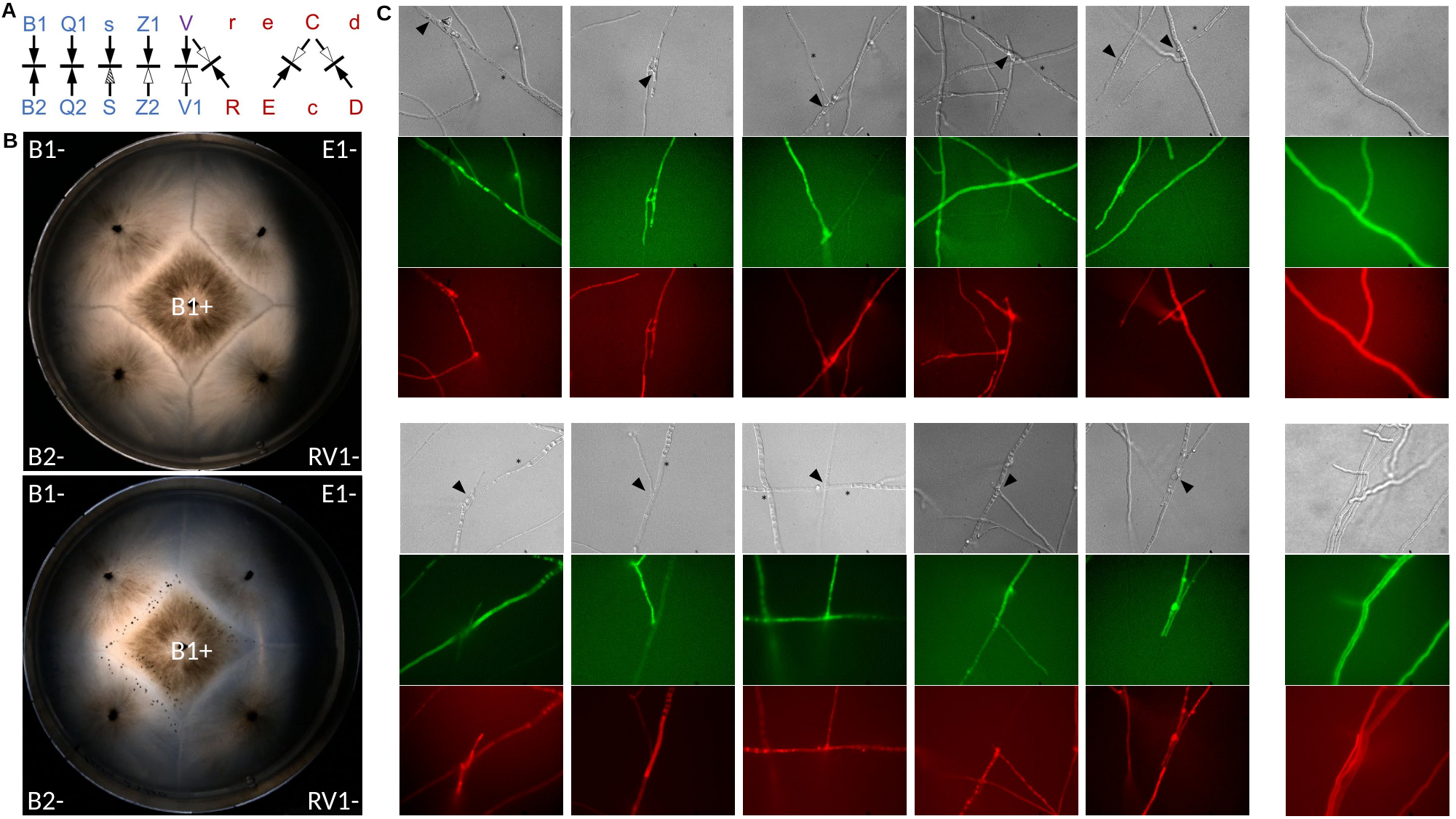

A
C
*
*
*
*
*
B
B1-
E1-
B1+
B2-
RV1-
*
*
B1-
E1-
*
*
B1+
B2-
RV1-

Supplement: S1 Fig — (A) The different genetically defined het systems of P. anserina are shown. Allelic systems are given in blue and non-allelic systems in red. het-V is involved both in allelic and non-allelic interactions and is given in purple. Opposing arrows represent an incompatible interaction. Full arrowheads indicate normal fertility, open arrowheads indicate sexual incompatibility. The arrow direction represents the direction of the cross, with the arrow pointing from the male parent to the female parent in the cross (for example for the V/V1 interaction, the diagram denotes that a male V1 x V female cross shows sexual incompatibility while the opposite cross is fertile). In the s/S interaction, the dashed arrowhead denotes the spore-killing reaction occurring in S male x s female crosses and leading to specific abortion of S spores. B. Barrage reaction (incompatibility) but lack of sexual incompatibility between B1 and B2. The strains of the given genotypes were confronted on corn meal agar and grown for 6 days in the dark (upper panel). The same plate was imaged again after a week under constant illumination (lower panel). Full incompatibility genotypes for relevant loci are as follows: B1: het-B1, het-c2, het-d3, het-e4, het-r, het-V; B2: het-B2, het-c2, het-d3, het-e4, het-r, het-V; E1: het-B1, het-c1, het-d3, het-e1, het-r, het-V; RV1: het-B1, het-c2, het-d3, het-e4, het-R, het-V1. Mating type is designated with—and + symbols. Note that fructifications (perithecia) form at the B1/B2 interface indicating the absence of sexual incompatibility in contrast to the C/E and RV1/rV interactions that show partial or total sterility respectively. C. Microscopic observation of the confrontation zone between a B1 strain expressing GFP and a B2 strain expressing RFP. The presumed anastomosis sites are marked by an arrowhead and lysed cell with an asterisk. In upper and lower right end panels, the confrontation zone between a B1 strain expressing GFP and a B1 strain expressing RFP sh [file pgen.1011114.s001.pptx]

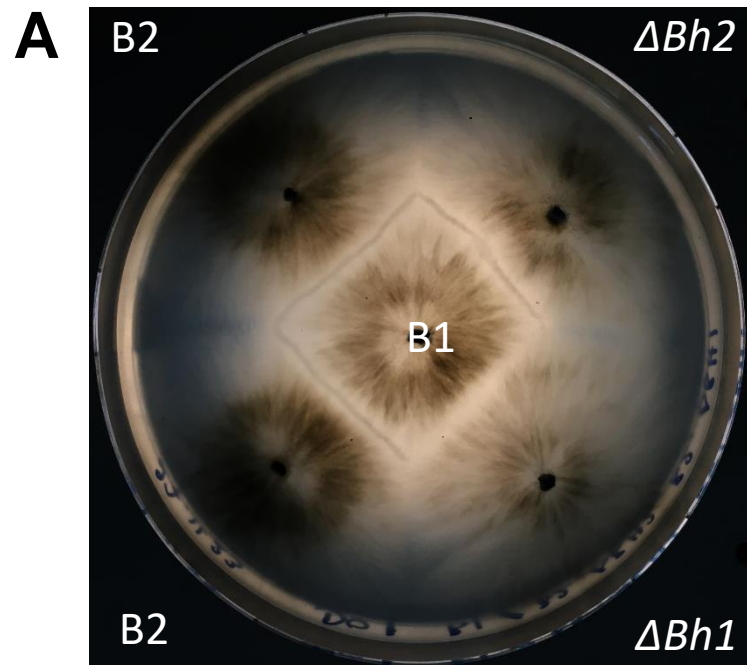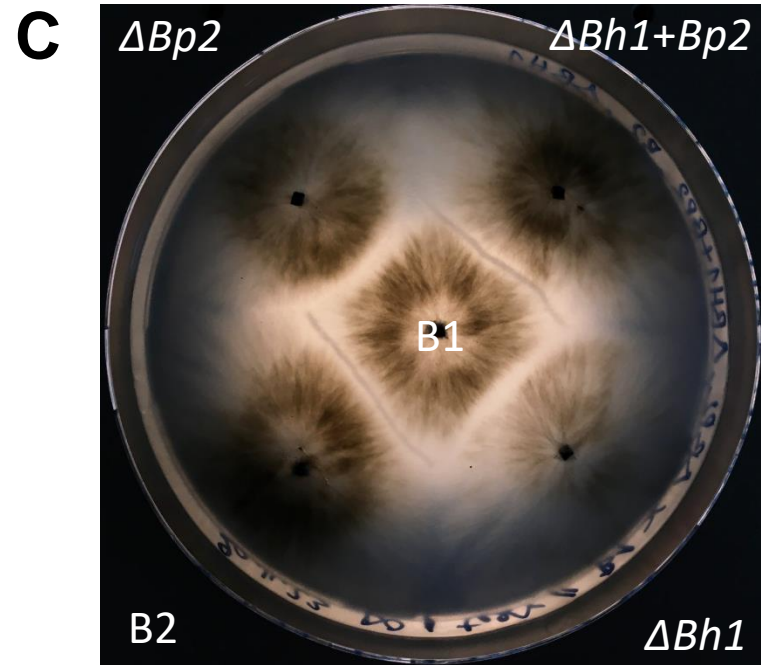

**B**

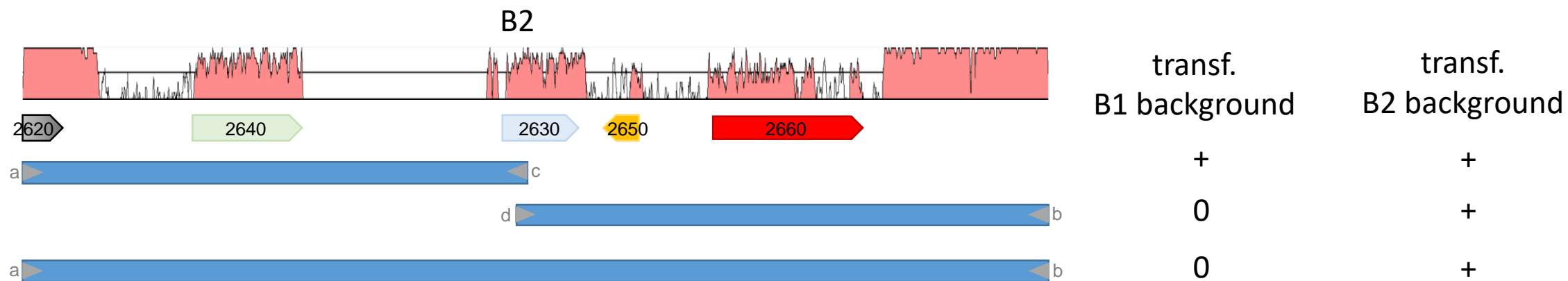

Supplement: S2 Fig — (A) Incompatibility phenotype of a ΔBh2 strain. ΔBh2 strains show a barrage reaction to B1. (B) Schematic summary of transformation efficiencies experiments of three fragments of the B2-locus region (fragments ab, ac, db). No transformants were obtained with fragments db and ab transformed into the B1 strain. (C) Bp2 determines B2-incompatibility. Strains deleted for Bp2 (ΔBp2) show no barrage reaction to B1 and ΔBh1 strains transformed with Bp2 produce a barrage reaction to B1. (PDF) [file pgen.1011114.s002.pdf]

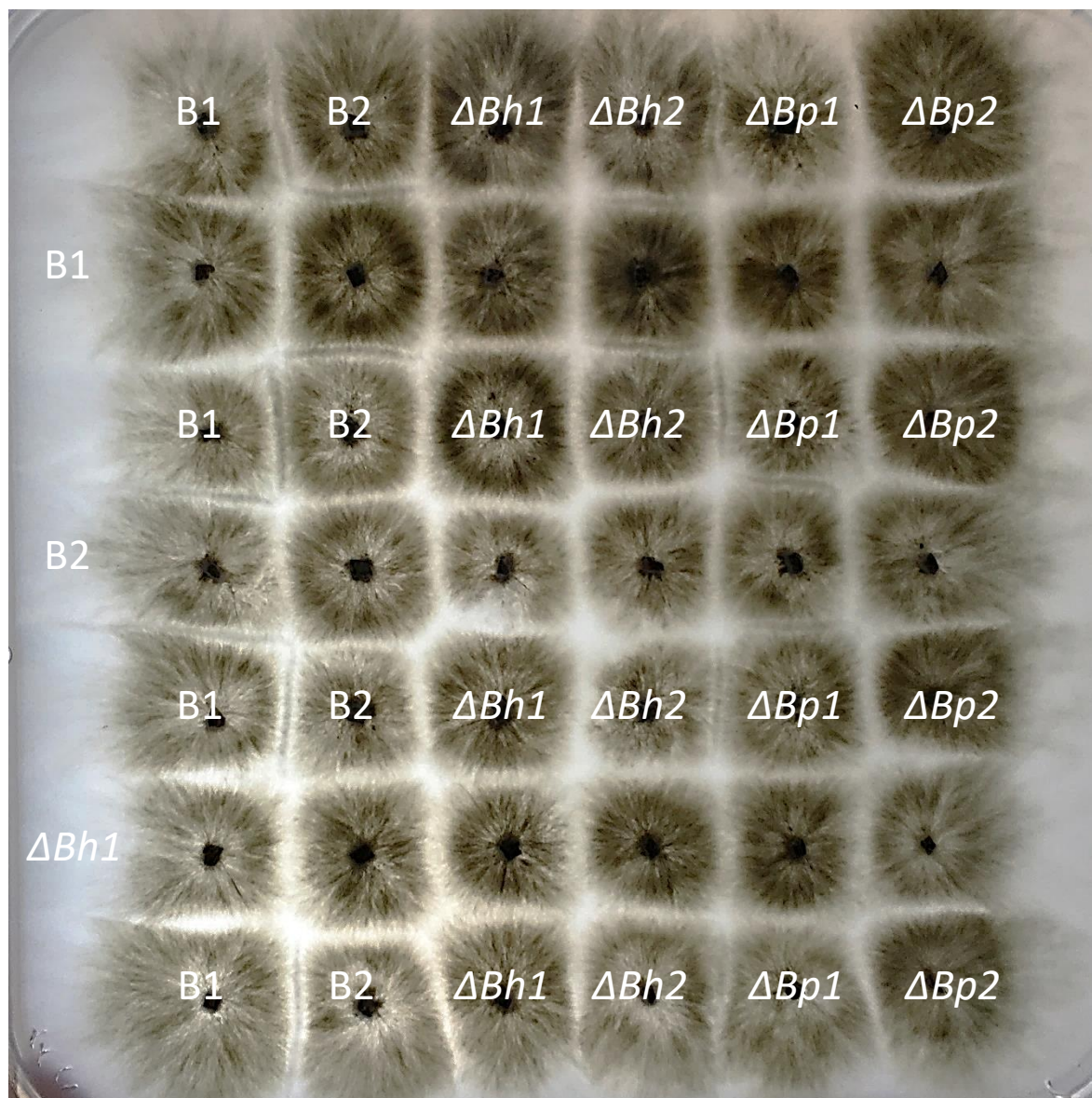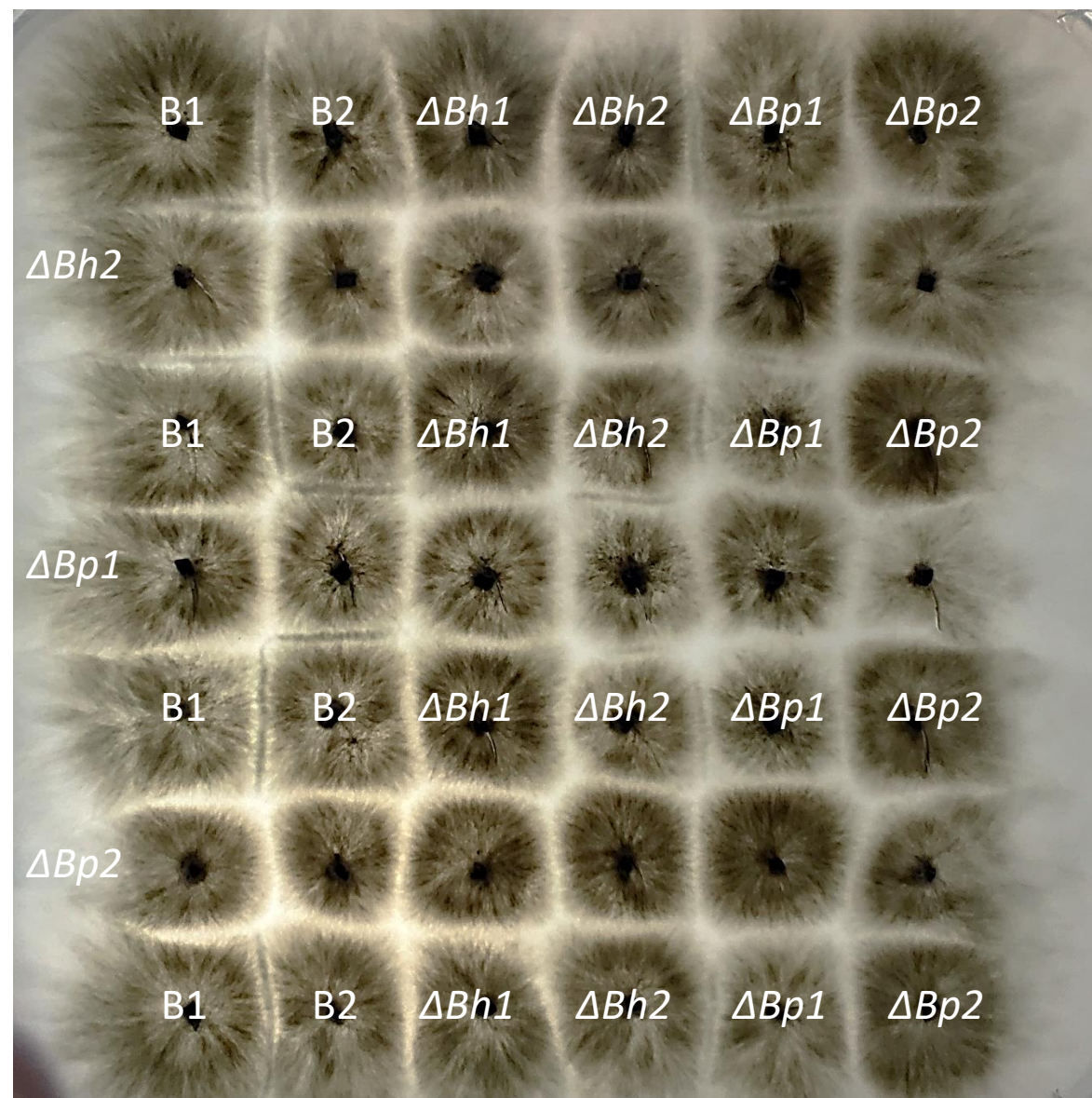

Supplement: S3 Fig — Strains with the indicated genotype were grown for 5 days on corn meal agar at 26°C. (PDF) [file pgen.1011114.s003.pdf]

**A**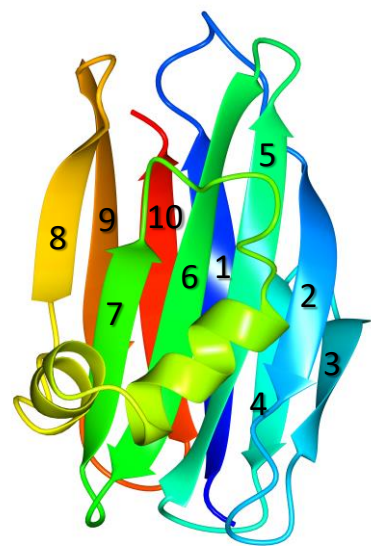

*Sclerotium rolf sii*  
lectin

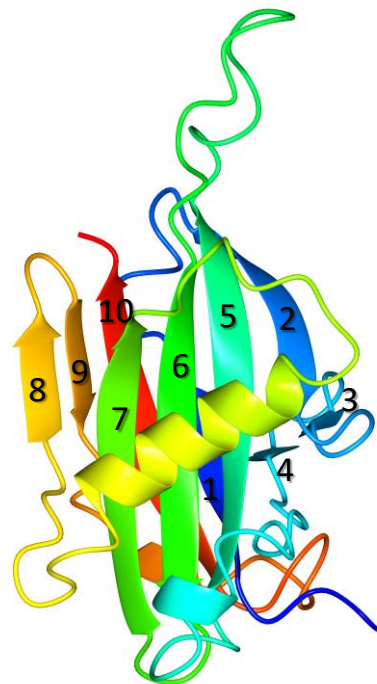

BH1 (445-610)

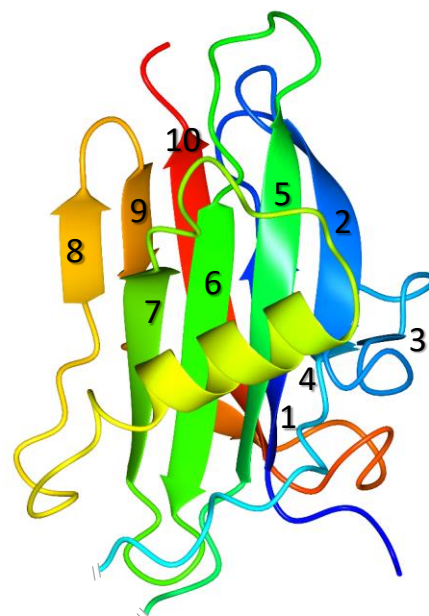

BH2 (451-627)

**B**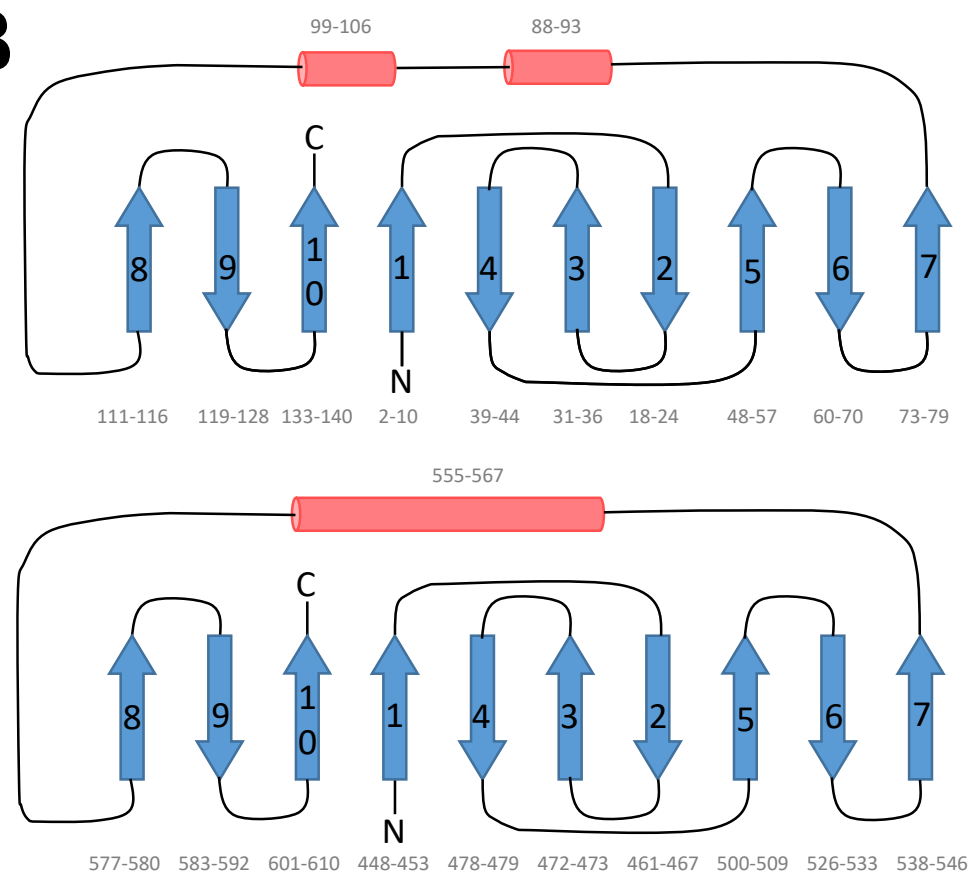**C**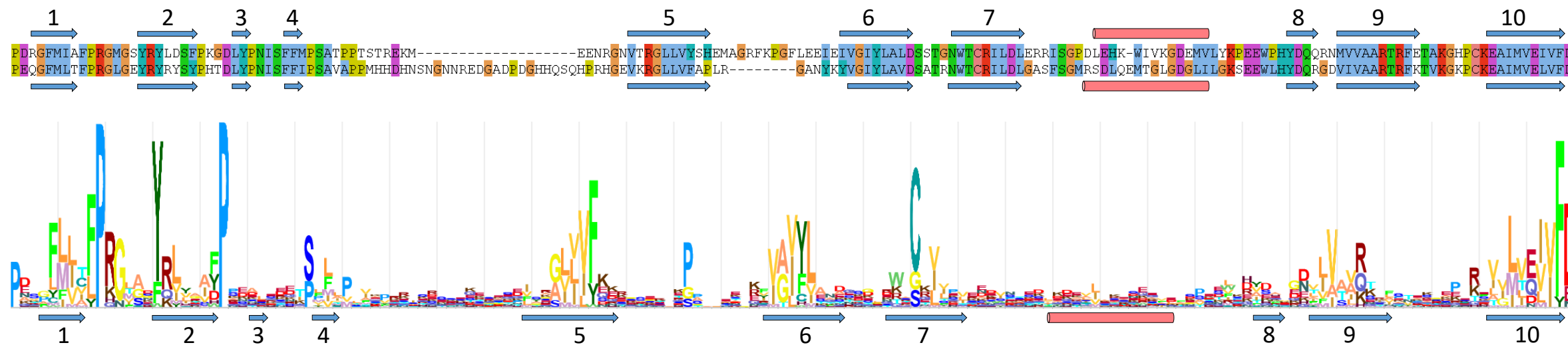

**D**

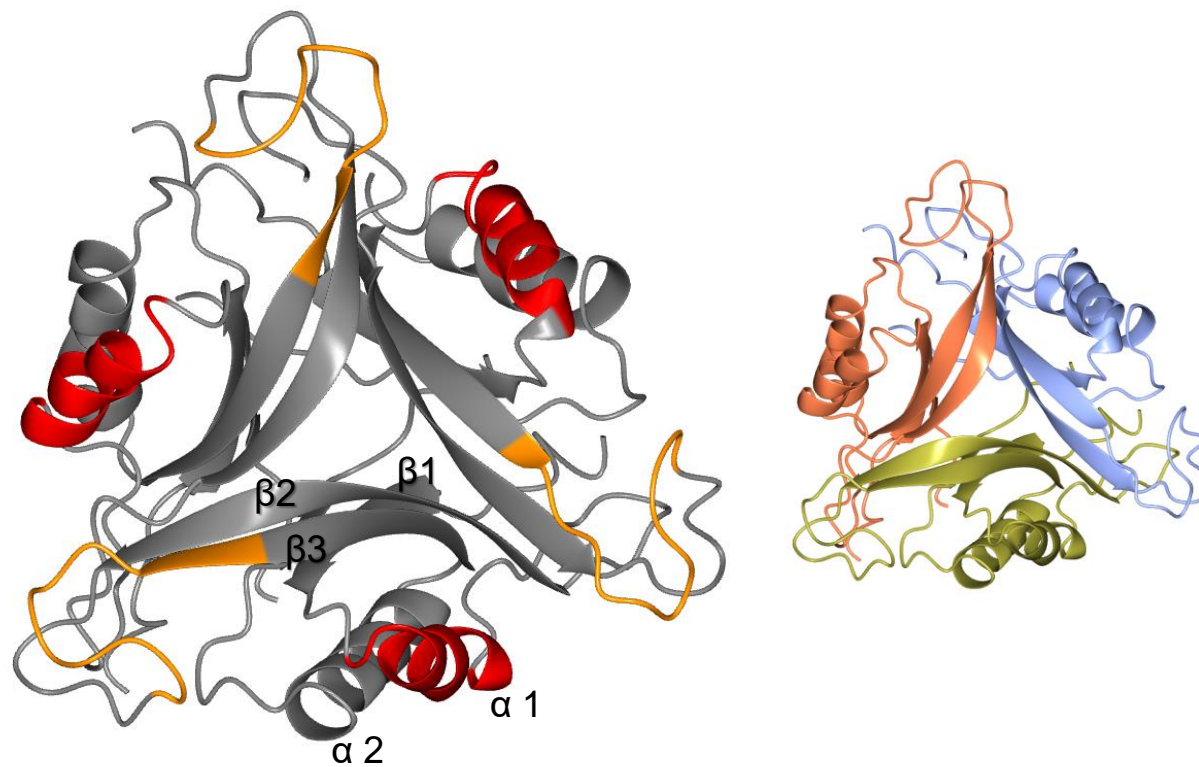

**E**

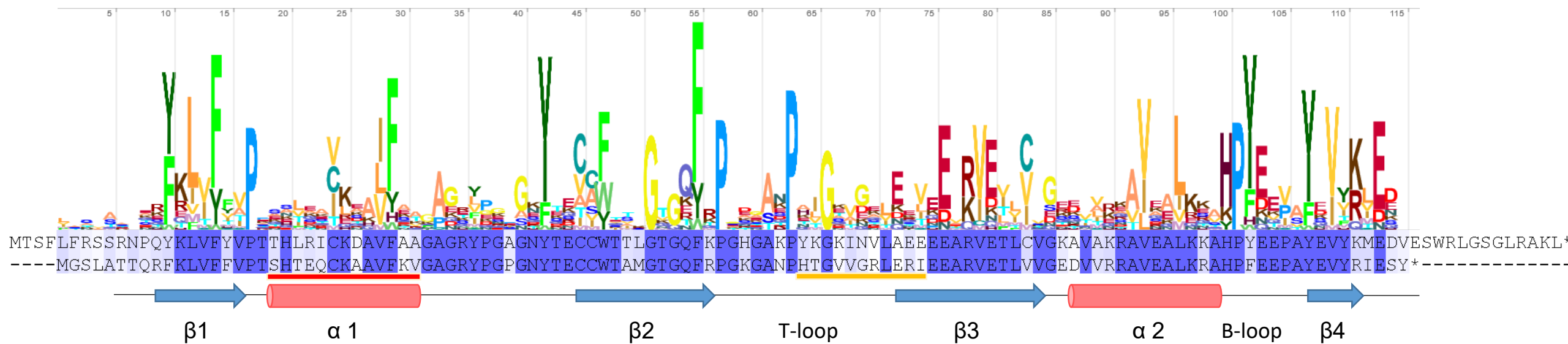

Supplement: S4 Fig — (A) AlphaFold models of the BH1 and BH2 lectin fold domains are given together with the structure of the Sclerotium rolfsii SRL lectin (pdb:2OFC). The β-strand regions are numbered from N- to C-terminus following the topology diagram given in B. In BH2 a large loop region between β-strand 4 and 5 was omitted. (B) Topology diagram of the SRL lectin (top) compared to the topology diagram derived from the BH1 lectin fold domain AlphaFold model (bottom). (C) HMMER consensus sequence of lectin fold domain of fungal BH-homologs. Above the consensus sequence, an alignment of BH1 and BH2 is given. Position of the predicted β-strands (blue arrows) and α-helix (red cylinder) are given both for the alignment and the consensus sequence. (PDF) [file pgen.1011114.s004.pdf]

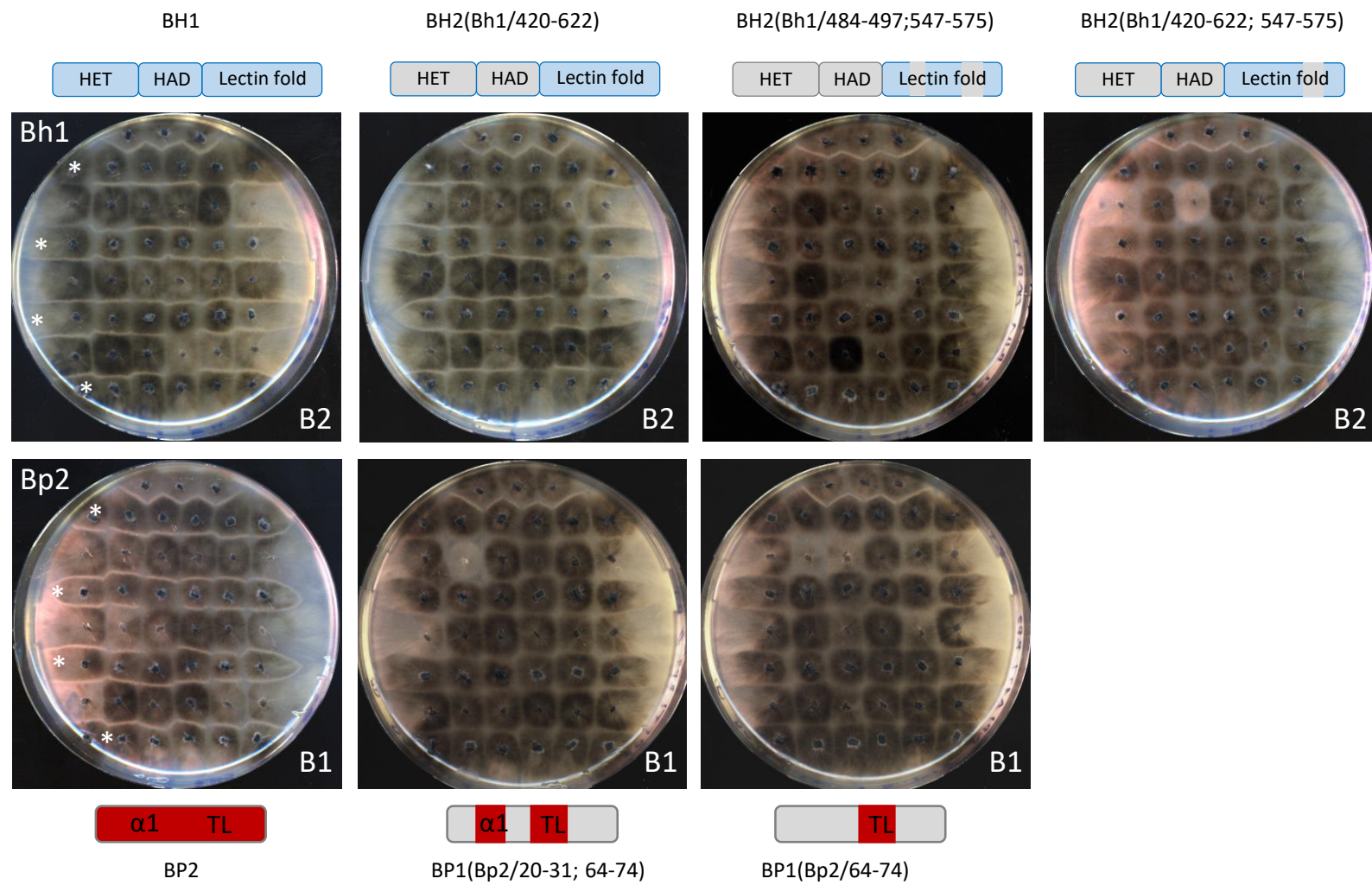

Supplement: S6 Fig — Upper panels, ΔB strains (lacking the entire B1 haplotype) were transformed with either Bh1 or the chimeric Bh2-derived alleles as given and individual transformants were assayed in barrage test in confrontation to B2 (lines of B2 tester strains are marked with an asterisk). The domain diagram of Bh1 and the chimeric alleles are recalled. Lower panels, ΔB strains (lacking the entire B1 haplotype) were transformed with either Bp2 or the chimeric Bp1-derived alleles as given and individual transformants were assayed in barrage test in confrontation to B1 (lines of B1 tester strains are marked with an asterisk). The domain diagram of Bh1 and the chimeric allele are recalled. On each plate, the upper three implants are positive control for incompatibility, that is B1 strains in the upper panels and B2 strains in the lower panels. (PDF) [file pgen.1011114.s006.pdf]

A

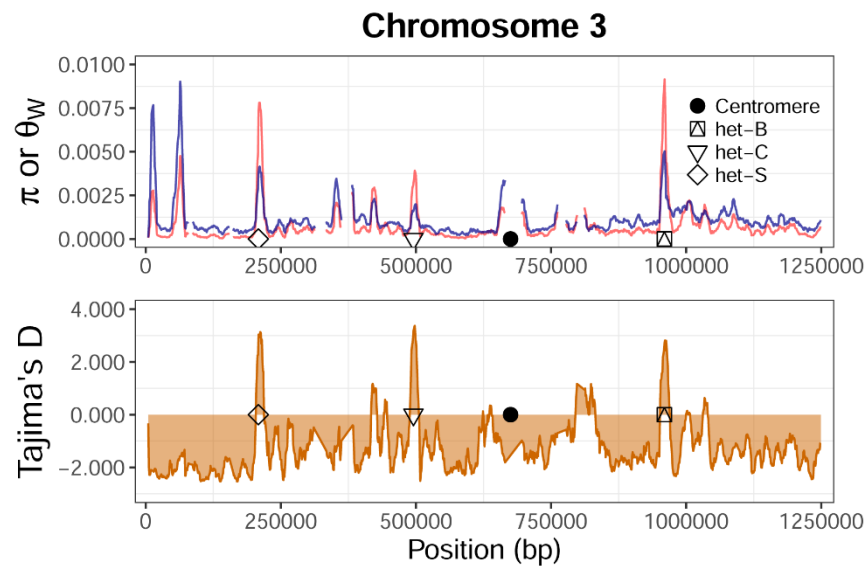

B

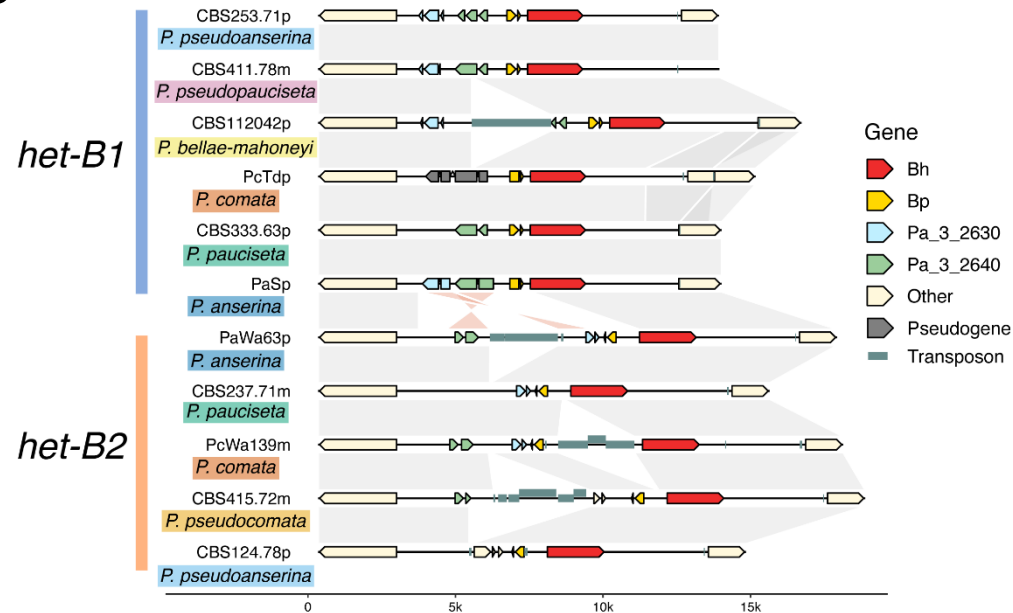

C

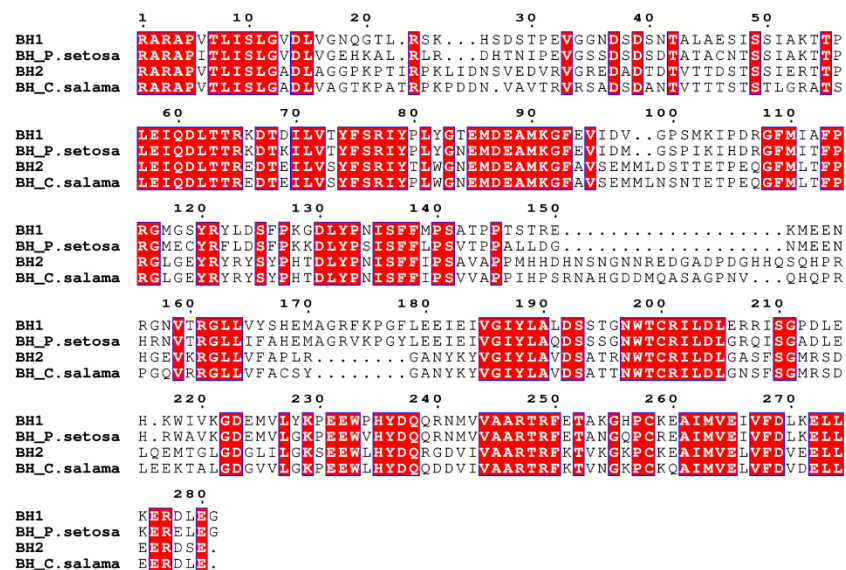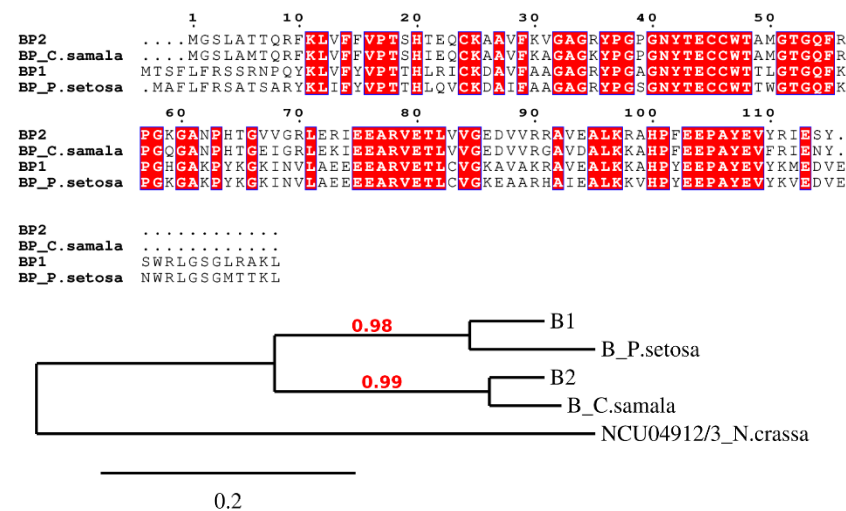

Supplement: S7 Fig — (A) Population genetic statistics in the Wageningen collection of P. anserina (1991–2016). Sliding-window analysis (10 kb-long with steps of 1 kb) of the first part of chromosome 3. The upper panel contains two metrics of genetic diversity: the pairwise nucleotide diversity π in red and Watterson’s theta θW in blue. The lower panel depicts the Tajima’s D statistic (orange). Symbols mark the position of the centromere and known het genes in the area. Data from Ament-Velásquez et al. (2022) [13],. (B) Architecture of the two het-B haplotypes is conserved across the P. anserina species complex. Same-sense alignments are represented with gray links, while inverted alignments are in light red. Gene exons are represented with boxes, with sense indicated by the orientation of their triangular side. The difference in gene structure between species might be due to sequencing or annotation errors, as well as real biological differences. (C) An alignment of BP1 and BP2 and their homologs in Cercophora samala and Podospora setosa is given together with an alignment of the lectin fold domain of BH1 and BH2 and their homologs from the same species. The phylogenic tree is based on the concatenated sequences of BP and the lectin fold domain of BH-homologs as given in the alignment. Sequences from N. crassa NCU04912 and NCU04913 are used as outgroup. (PDF) [file pgen.1011114.s007.pdf]
