# Supplementary material for: het-B allorecognition in Podospora anserina is determined by pseudo-allelic interaction of genes encoding a HET and lectin fold domain protein and a PII-like protein
Source: PLoS Genet. 2024 Feb 12;20(2):e1011114. doi: 10.1371/journal.pgen.1011114 (PMC10890737; doi:10.1371/journal.pgen.1011114)
Supplement: S5 Fig — (A) Sequence alignment of BP1 and BP2 and a bacterial PII-like protein, AZV25447.1 from Pseudomonas syringae. (B) AlphaFold model of BP2. The same model is given twice, on the left to highlight polymorphic regions (the polymorphic T-loop and α1 regions highlighted in orange and red) and on the right, with a different coloring for each subunit to illustrate the predicted trimeric structure. (C) A HMMER consensus sequence for fungal BP-homologs is given together with the sequence alignment of BP1 (upper sequence) and BP2 (lower sequence). The polymorphic T-loop and α1 regions highlighted in the AlphaFold model are underlined in orange and red in the two alignments. (PDF) [file pgen.1011114.s005.pdf]

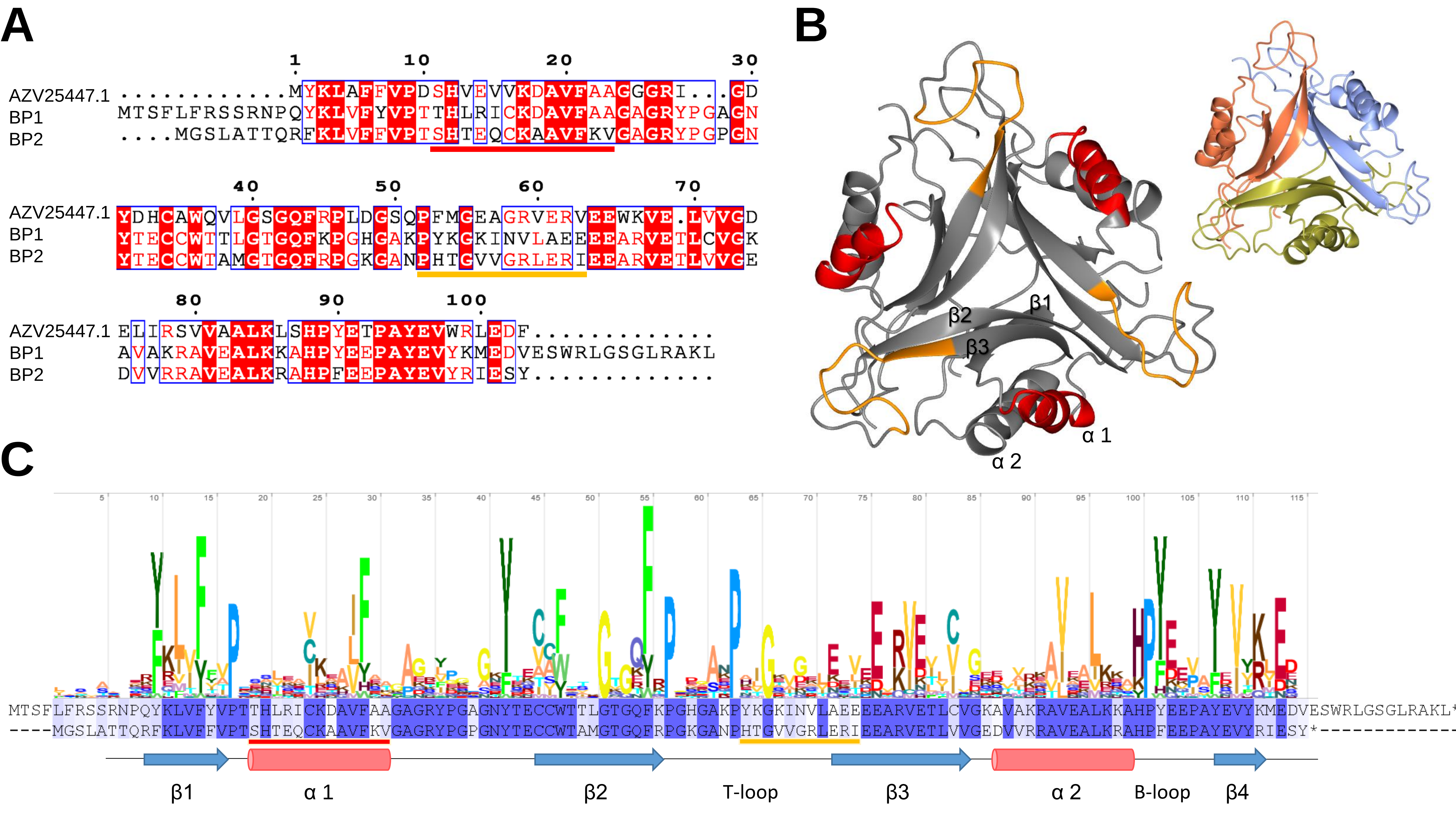
A
1 10 20 30
AZV25447.1 .............MYKLAFFVPDSHVEVVKDAVFAAGGRI...GD
BP1 MTSFLFRSSRNPQYKLVFYVPTTHLRICKDAVFAAGAGRYPGAGN
BP2 ....MGSLATTQRFKLVFFVPTSHTEQCKAAVFKVGAGRYPGPGN
40 50 60 70
AZV25447.1 YDHCAWQVLGSGQFRPLDGSQPFMGEAGRVERVEEWKVE.LVVGD
BP1 YTECCWTTLGTGQFKPGHGAKPYKGKINVLAEEEEARVETLCVGK
BP2 YTECCWTAMGTGQFRPGKGANPHTGVVGRLERIEEARVETLVVGE
80 90 100
AZV25447.1 ELIRSVVAALKLSHPYETPAYEVWRLEDF...............
BP1 AVAKRAVEALKKAHPYEEPAYEVYKMEDVESWRLGSGLRAKL
BP2 DVVRRAVEALKRAHPFEEPAYEVYRIESY...............
B
β2
β1
β3
α 1
α 2
C
MTSFLFRSSRNPQYKLVFYVPTTHLRICKDAVFAAGAGRYPGAGNYTECCWTTLGTGQFKPGHGAKPYKGKINVLAEEEEARVETLCVGKAVAKRAVEALKKAHPYEEPAYEVYKMEDVESWRLGSGLRAKL*
----MGSLATTQRFKLVFFVPTSHTEQCKAAVFKVGAGRYPGPGNYTECCWTAMGTGQFRPGKGANPHTGVVGRLERIEEARVETLVVGEDVVRRAVEALKRAHPFEEPAYEVYRIESY*------------
β1
α 1
β2
T-loop
β3
α 2
B-loop
β4

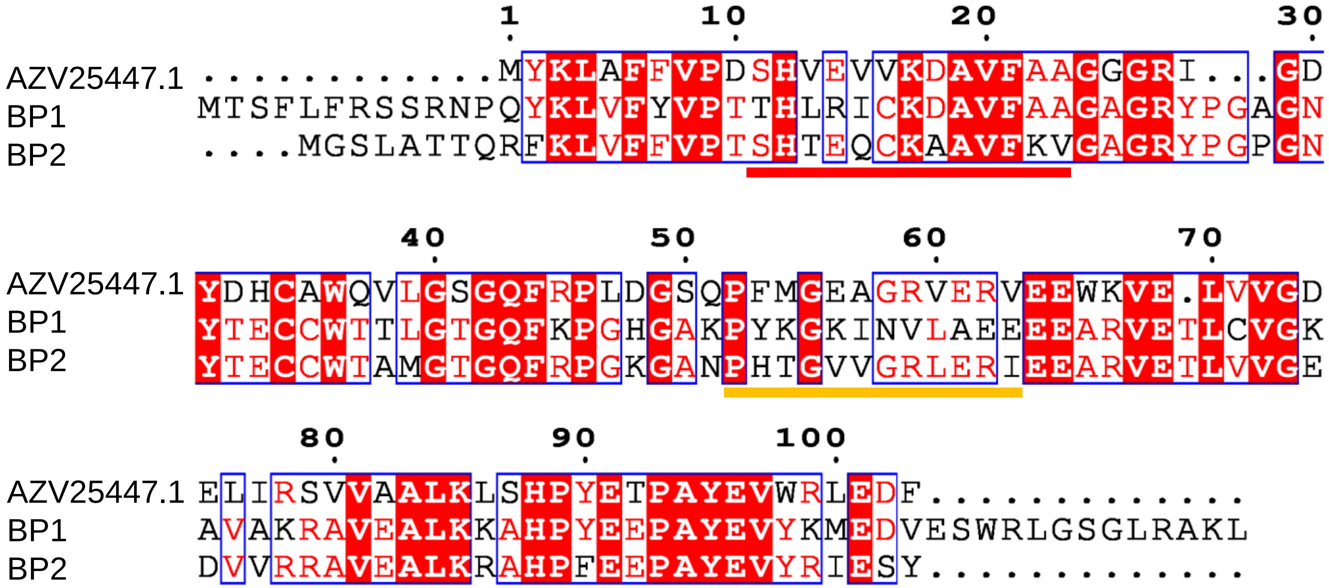
1 10 20 30
AZV25447.1 .............MYKLAFFVPDSHVEVVKDAVFAAGGGRI...GD
BP1 MTSFLFRSSRNPQYKLVFYVPTTHLRICKDAVFAAGAGRYPGAGN
BP2 ....MGSLATTQRFKLVFFVPTSHTEQCKAAVFKVGAGRYPGPGN
40 50 60 70
AZV25447.1 YDHCAWQVLGSGQFRPLDGSQPFMGEAGRVERVEEWKVE.LVVGD
BP1 YTECCWTTLGTGQFKPGHGAKPYKGKINVLAEEEEARVETLCVGK
BP2 YTECCWTAMGTGQFRPGKGANPHTGVVGRLERIEEARVETLVVGE
80 90 100
AZV25447.1 ELIRSVVAALKLSHPYETPAYEVWRLEDF..............
BP1 AVAKRAVEALKKAHPYEEPAYEVYKMEDVESWRLGSGLRAKL
BP2 DVVRRAVEALKRAHPFEEPAYEVYRIESY..............
