# Supplementary material for: het-B allorecognition in Podospora anserina is determined by pseudo-allelic interaction of genes encoding a HET and lectin fold domain protein and a PII-like protein
Source: PLoS Genet. 2024 Feb 12;20(2):e1011114. doi: 10.1371/journal.pgen.1011114 (PMC10890737; doi:10.1371/journal.pgen.1011114)
Supplement: Attachment — Submitted filename: reponse letter.docx [file pgen.1011114.s015.docx]

Dear Editors,

Please find hereafter our response to the reviewer’s comments and the description of the changes made to the manuscript.

We would like to express our thanks to the reviewers and editors for their interest in this work and their amiability.

For clarity, the original comments from the reviewers have been copy-pasted anew below and are given in blue and italicized.

We provide a version of the manuscript with highlighted changes. A few additional typing errors were spotted by us after the submission and have also been corrected. Fig.1, 5, S1 and S6 have been minimally modified. In Fig. 5 and S6, the domain designation “had” was changed to “HAD” for uniformity as well as “lectin-like” to “lectin fold”. In Fig.1 panel C, “het-B2” was changed to “B2”. In S1 Fig., there was an error in the image panel. The panel in visible light on the the top right corner did not match the GFP and RFP images. This was corrected.

*Reviewer #1: (…)
Perhaps the one weakness, which is acknowledged by the reviewers, was not providing experimental evidence of physical interactions between the Bh1 and Bp2 proteins (with one control presumably to see no interaction between Bh2 and Bp1?).*

We agree with the reviewer that determining whether (or not) Bh and Bp interact physically is important to gain further insight into the mechanistic basis of this system. Towards that end, we have obtained functional epitope tagged versions of both Bh and Bp that could potentially be used to analyze their interaction using immunoprecipitation approaches. The optimal experimental setting to perform such an experiment would be to use strains that co-express Bh1 and Bp2. But this approach is hindered by the fact that this co-expression leads to cell death. We now know of two ways that allow Bh1/Bp2 co-expression without the associated induction of cell death. One involves the use of a point mutant of the HET-domain of Bh affected for a residue that alleviates the NAD-hydrolyzing activity (that we found is responsible for RCD), the second involves the use of extragenic suppressors (in genes modA and modB) that suppress HET-domain associated RCD. Both means allow for co-expressing of Bp2 and Bh1 without induction of RCD. These findings are however not published at present and are intended to be part of a separate exploration of HET-domain function in RCD. This is the reason why we have not engaged in analysis of the Bp/Bh interaction in the present study. We hope that the reviewer will agree that despite this weakness, the present study remains of interest as a waypoint towards the characterization of HET-domain associated RCD.

*Minor points:
Line 74: remove italics from ‘and’.
Line 231: capital P for ‘Pezizomycotina’.
Line 243: elsewhere these higher taxonomic groups are in italics, so probably good to check the format and be consistent throughout.
Line 249: remove italics from ‘sp.’.
Line 299: add italics to ‘V’.
Line 390: ‘molecule’ (or ‘molecular’).
Line 481: ‘has’ to ‘as’.
Line 510: remove italics from the ends of ‘NruI’ and ‘BglII’, and change ‘3,6’ to ‘3.6’.
Line 554: two times change ‘pb’ to ‘bp’.
References: the journal will not reformat these or edit them, so if you want consistent formatting and, e.g. italics on species or gene names, this could be done now.
Line 1029 and 1040: ‘HMMER’.*

We thanks the reviewer for the careful reading and have corrected of these mistakes.

*Reviewer #2:(…)
I have only some minor comments:
For better readability I suggest, to explain the abbreviations HET- and TIR-domain in the abstract and Introduction. Not everybody of the readers is familiar with the paper of the author from 2007 (reference 42), where these domains are explained.*

The abbreviation for the TIR domain is spelled out both in the abstract and introduction section (L23 and L93. As for the HET domain, we hope it is obvious that the name derives from the fact that it was first identified in *het*-genes (L92).

*Further, fig. 2 could be omitted, since the fig. does not really contribute to the understanding of the genetic differences of the het loci.*

If possible we would like to retain that figure. We respectfully disagree with the reviewer and feel that the figure allows to appreciate the extend of sequence divergence between the two haplotypes such as the change in relative gene orientation and presence of completely distinct regions (for instance upstream of *Pa_3_2630* in B2).

*Minor points:
Give references for Podospora didyma (line 238)*

A reference to a recent phylogeny paper (Hensen et al, 2023, ref. 71) was added for *P. didyma*.

*Reference titles are given in capital letters or in lowercase letters*

We thanks the reviewer for the careful reading.

*Reviewer #3: (…)


L82, The reference to Figure 1A here seems out of place.*

This is correct. Thank you. The reference should be to S1 Fig. This is now corrected. *L160, pb should be bp.*

This mistake was corrected.

*L181. I hadn’t heard of the PII protein family before, and I found it frustrating that I didn’t know how to pronounce this name when reading the text (I wondered was it P.I.I. or P2 ?). From a literature search, I found the latter is correct and the name is often written as P(II) or with the “II” as a subscript. The name is trivial because it originates from the initial identification of the protein in 1969 as the second peak eluting from a gel filtration column, but changing the text to say P(II) or using a subscript would make it more obvious how to pronounce this name.*

We have faced the same issue, we admit. We however chose to adhere to the “PII”, the writing as currently used by Khaled Selim (University of Freiburg), one of the most active researcher in that area.  *L231. Pezizomycotina should have uppercase P.*

This was corrected. *L286. Need a reference for the previously published sequencing data.*

The reference was added (Ament-Velásquez et al. 2022, ref.13). *L365-373: Lectins bind glycans, so is it known whether the PII proteins are glycoproteins?*

We are sorry but we are not aware of reports reporting on the glycosylation statues of PII proteins. This field is new to us and this information might exist but we are unable to comment more. *L418, typo – should be ”mesosynteny”.

L894, typo – should be “shortened”.

L974, typo – should be “systems”.*

These mistakes have been corrected.

*L974-982 and L73. Figure S1A summarizes a lot of information, but the only reference cited as the source of this information is Bernet 1967 (ref. 22). Is this correct? Incredible if true!*

The Bernet 1967 reference is indeed the source for the information in S1 Fig. The short four page long 1967 article synthesizes the entire genetic analysis of the *P. anserina het* systems and cites a longer manuscript corresponding to the thesis of J. Bernet (ref. 24). While the Bernet 1967 paper is directly available on the internet, the longer thesis manuscript is not. We cannot but be agree with the reviewer that the breadth of this genetic work is remarkable. It has served as the stepping stone for our labs’ efforts for decades.
